# Treatment of Chronic Stomach Troubles by Irrigation

**Published:** 1888-02

**Authors:** Herman E. Hayd

**Affiliations:** M. D., M. R.C.S., Eng., Buffalo, N. Y.; No. 78 Niagara street


THE BUFFALO MEDICAL AND SURGICAL JOURNAL

VOL. XXVII. FEBRUARY, 1888. No. 7

### Original Communications.

### *TREATMENT OF CHRONIC STOMACH TROUBLES BY IRRIGATION.*

By HERMAN E. HAYD, M. D., M.R.C.S., Eng., Buffalo, N. Y.

There are few affections which render life more miserable than chronic dyspepsia, associated, as it is, with so many varied and troublesome phenomena. Moreover, no class of diseases for which so many different remedies have been given, including acids, alkalies, bitter infusions, digestive agents, astringents, tonics, and what not. Nor can it be said that any one or all of these agents have given anything but temporary relief. Therefore, the greatest patience on the part of the physician, and no less forbearance from the patient, is necessary in treating these very troublesome diseases.

During the last decade, a most important as well as satisfactory method of treating these affections has been largely used in Germany, viz., irrigation of the stomach; and, by means of it, the most inveterate cases have been permanently cured. For the past three or four years, I have resorted to irrigation very frequently, and have invariably cured the most obstinate forms of chronic dyspepsia, and have relieved materially many of my patients by three or four washings, when they would not submit to a continuance of the treatment.

Unfortunately, this procedure requires much moral courage. Although not accompanied by any pain, yet it is a very disagreeable operation, but becomes less objectionable after three or four seances. At first, the gagging and retching are considerable, and sometimes slight capillary hemorrhages occur, which are apt to alarm very sensitive people. Add to this, much depression, and even shock, during the first operations; and we will not be surprised to see how reluctantly some submit to this treatment. Yet, as a general rule, the fear and dread is largely imagination; so that, after the first treatment, the patient swallows the tube without much trouble, and submits to the operations very willingly.

The instrument which I have used is a very simple affair, and may be obtained for a few dollars. It consists of an œsophageal tube, about one inch in circumference, and about forty inches in length. To its free end is attached a piece of rubber tubing, about four feet in length, and to it an ordinary-sized funnel.

The patient is seated upon a chair, with the head well thrown backwards. The instrument is dipped into warm water, and thoroughly softened, and then annointed with some unctuous material, preferably glycerine. The index finger of the left hand is placed firmly upon the base of the tongue, and the instrument—the point of which is slightly bent—is held loosely in the right hand, and passed into the pharynx, and directed along its posterior wall. When the opening into the œsophagus is reached, the admission of the instrument is obstructed from spasm. The patient is now directed to swallow; and these efforts, together with slight pressure on the tube, forces it into the œsophagus, and then its onward course is easily accomplished.

Much can be done to obviate the difficulties attending the first operations, by painting the pharynx and soft palate with a four per cent. solution of cocaine. This renders the parts less susceptible to irritation and reflex action; consequently, the gagging and discomfort is much lessened.

I prefer to have the patient see me in the morning, about an hour and a-half or two hours after having taken a cup of beef tea or warm milk; in fact, try to have the stomach empty and free of all undigested food, as these lumps are apt to get into the eye of the tube, and give rise to no end of trouble. About two gallons of water is used for each operation, and the temperature of it is about 106° at first, and is gradually increased to 120° F. The water is slowly poured into the funnel, well elevated above the patient's head, and just as soon as feelings of discomfort and distension are complained of, the tube is quickly depressed, and by siphonage the fluid runs out. The ablution is continued until the escape fluid is clear and sweet. At first, the stomach will only tolerate about a quart, but, after a few washings, much more fluid can be introduced. This is a very important feature, because, by thoroughly distending the stomach—not to over-distension—the organ is rid of all offending and irritating mucus, and circulation and absorption is made more active. It is well to add some alkali, say sodæ bicarb., ʒj., and sodæ bibor., grs. xx., to each quart of water. The former renders the mucus less tenacious, while the latter is slightly astringent, stimulant, and antiseptic. Again, boracic acid can be used with advantage. If gastralgia or enteralgia have been distressing symptoms, a-half a drachm of chloroform, or one drachm of spts. ether. chlor., is a very valuable addition to this solution.

CASE I.—Mrs. D., *aet.* twenty-seven, consulted me on August 18, 1885. Has two children; youngest, eighteen months old. Always enjoyed good health, was vigorous, cheerful and light-hearted; weighed 176 pounds.

About fifteen months ago, she went to a dinner-party, and ate very heartily of many rich dishes. That night, she was seized with a violent attack of acute indigestion. Since that time, has complained of more or less uneasiness and distress in the pit of the stomach, and particularly after taking food. Flatulence, drowsiness, mental depression, often approaching melancholia, severe headache, especially over the eyebrows and through the temples, obstinate constipation, etc., etc.; heart-burn, for which she had taken enormous quantities of bicarbonate of soda; nausea, and even vomiting, at the sight of food. She lost flesh rapidly, the various functions of the body were disturbed and irregular, and, finally, she became a confirmed invalid. I was called to the Tifft House one night, and met the patient for the first time. She was on her way home to Indiana, after having been in New York for some weeks under the care of a specialist. She was a pale, anæmic brunette, with large frame and big bones, and weighed one hundred and twenty-six pounds. She had been vomiting for several hours, and was quite exhausted. The vomit contained much thick, stringy mucus. The tongue was large, swollen, slightly coated with a greyish fur, and traversed laterally and horizontally with deep fissures and cracks. The papillæ about the tip and edges were prominent, red, and angry-looking. Bowels constipated; pulse ninety-six, soft, regular and compressible. I prescribed the usual remedies: mustard plasters to the pit of the stomach, broken pieces of ice, small doses of calomel, and rest. In a few days, I advised irrigation, which she hesitatingly submitted to; but finally consented, since all other remedies ever employed did her little or no good. I washed the stomach three times a week, and prescribed a powder, sodæ bicarb., grs. v., bismuth trisnit., grs. ii., hydrarg. chlor. mitis, grs. ii., *ter in die ante cibum*, also peptonized milk, broths, and other easily-digested and assimilable foods. She was under my care for eight weeks, and, during this period, I washed the stomach eighteen times. The improvement in her general condition was very marked from the first operation. She had less distress with her food, little or no flatulence, and gained rapidly in flesh and strength, which improvement continued uninterruptedly. In these eight weeks, she gained thirteen pounds. I saw her again last October, when she weighed 158 pounds, and *felt very well*. She still exercises care with her diet.

Case II.—Mrs. W., *aet.* fifty-two; mother of four children; youngest, nineteen. Diagnosis—hysterical œsophagismus, associated with chronic dyspepsia. Patient has lost much flesh, is nervous and excitable, and at times exceedingly irritable.

For a number of years, has complained of various dyspeptic symptoms, but that which distressed her most was flatulence, accompanied with water-brash. After a time, nausea, and then vomiting upon the slightest ingestion of food. For the past six months, the emesis has not been associated with any depression. The food was simply regurgitated into the mouth in a semi-digested state, and spit out. She says that it is impossible to swallow any solid food—it sticks in her throat. For months, she had lived upon milk, broths, soups and liquid food of various kinds. Upon examination with a flexible bougie, a definite stricture was felt about the middle third of the œsophagus, which gentle pressure on the instrument, and a little time, overcame. The stomach was much dilated, and when a pint of water was poured into it, its lower border could be traced two inches and a-half below the umbilicus.

Lavage was advised, and the first washing brought away a surprising amount of an exceedingly fetid fluid, which contained much thick, stringy mucus. The operation was repeated six times, with considerable benefit. She left the city unexpectedly, so I was not enabled to follow up the treatment. Yet I had every hope of curing her. Moreover, she felt assured of her improvement, and intended to have her family doctor continue the treatment.

Case III.— Miss G., *aet.* twenty-five. Diagnosis— gastric ulcer. For three years, had suffered much from pain in the pit of the stomach, and especially upon taking food. About a year ago, this pain was very severe, and was often accompanied by vomiting. In the ejecta, she frequently saw blood. Is pale, anæmic, and complains of much tenderness upon palpation in the espigastrium.

As she had been under the care of various physicians, and had taken nearly every drug in the drug-shop, as she said, I advised her to submit to irrigation of the stomach. She did so without much persuasion, although at first I was reticent in using this means, lest I might perforate the stomach. But after having read an article in the *Medical Record* by Dr. Kinnicutt, who had treated many cases with great benefit, I concluded to make the trial with caution. At first, I only introduced a pint of water, and withdrew it, then repeated the procedure four times, and each time used about the same amount of water. To the last pint I added a-half a drachm of bismuth trisnit., well rubbed up with glycerine. The return fluid was slightly stained with blood. She called again in three days, and reported that she had suffered less pain than for months past. I repeated the washings eleven times, extending over a period of eleven weeks. She gained flesh and strength, muscles became firm and well-nourished, appetite returned, and she suffered little or no pain. Patient is still under observation.

Case IV.—Jas. S., *aet.* twenty-eight; engineer. Diagnosis—chronic dyspepsia; principal and most annoying symptom, intense vertigo; tongue small and contracted, very red, irritable, and angry-looking, and traversed by deep, irregular fissures. Had lost flesh and strength, and was compelled to give up his job, not so much from physical weakness, but on account of the awful responsibility of his position, as these attacks of dizziness often came on while in the engine, when he was forced to yield his position to his fireman.

The first washing brought away a brownish-yellow, foul-smelling fluid, abundantly mixed with mucus and tinged with blood. Upon each subsequent seance, the odor was less marked, and the color of the return fluid clearer, and mixed with less mucus. I washed the stomach twenty-four times, extending over a period of thirteen weeks. During this time, he gained twenty-two pounds. His nervous symptoms and vertigo entirely disappeared. The last treatment I gave him was in April, 1887, and since that time I have seen him frequently, but never had occasion to prescribe for him.

In this paper, I have purposely cited cases in whom different dyspeptic symptoms were especially distressing and annoying. I might say that the after-treatment, or, at least, the medical treatment, during the period of irrigation was practically the same. A carefully-regulated diet, plenty of open-air exercise, and occasionally a dose of pil. col. co., or a small dose of calomel, to relieve the obstinate constipation seen in most of these cases.

Much more could be written upon this subject, and other cases recorded, but it would only be to reiterate the same success, when the treatment was honestly carried out, as resulted in the four reported cases. Moreover, it is surprising to see the unanimity of feeling, and to read the gratifying reports in nearly every medical journal, of similar successes, by every observer who has given this method a fair trial. Some have used it with benefit in the treatment of cholera infantum, for the pains of cancer, for ulcerations, acute or chronic, for extreme dilatation of the stomach, with atrophy or hypertrophy of its walls—in fact, for every known stomach trouble.

No. 78 Niagara street.

